# Materials for Space Exploration Take a Giant Leap

**DOI:** 10.1021/acscentsci.3c00376

**Published:** 2023-04-07

**Authors:** Prachi Patel

“Also, I have duct tape.... Even NASA can’t improve on duct tape,” says Mark Watney, an astronaut stranded on the Red Planet in the sci-fi novel *The Martian*. Watney uses this unassuming material to seal a hole in his helmet and patch a large hole in his habitat.

Turns out, NASA astronauts do use duct tape for repairs in a pinch. But space is an unforgiving environment where things can go horribly wrong. For a voyage to Mars—which NASA is eyeing after putting humans on the moon again in 2025—much will rest on advanced materials that can withstand the rigors of space to safely get astronauts to their destination and back. And because it can take more than a decade to develop and certify a new material for spaceflight, agencies need to decide on future missions' key materials today.

The agency’s budget for human space exploration is over $7 billion for 2023. But costs quickly add up. “Just the fuel cost of sending a person to Mars is astronomical,” says Gregory M. Odegard, a materials scientist at Michigan Technological University. “It takes $1 million worth of fuel per pound of cargo to send a person there. People need a lot of stuff to survive on Mars, and before you know it, the payload becomes massive.”

To save costs, engineers need to make both spacecraft and their cargo out of smart materials that can multitask—materials that are not just lightweight but also superstrong, temperature and radiation resistant, and even capable of transforming in response to their environment. That’s why researchers are experimenting with emerging materials and bestowing tried-and-tested components with new properties. “Much of our job is to take the best and make it better,” says Stephanie Vivod, a chemical engineer at the NASA Glenn Research Center. “But we also have opportunities to create things that have never even existed.”

## LIFTOFF

Even by spacecraft standards, the launch vehicle for the Artemis lunar mission is astounding in heft. Taller than the Statue of Liberty and weighing 2,600 tons (t) when fueled, it can propel more than 27 t of cargo to the moon. A Mars mission will need an even bigger payload. Each kilogram that can be shaved off saves fuel needed to fight gravity at liftoff.

Materials technologies have taken giant leaps in the 50 years since NASA’s Apollo missions. The polymer composites industry was in its infancy back then, and spacecraft parts were built with lightweight aluminum panels and honeycomb structures, plastics, and early composites. Many of those materials have been replaced with even lighter aluminum–lithium alloys and composites of carbon fibers impregnated with resin.

NASA now wants to triple the strength of those carbon fiber composites. The components going into rockets, fuel tanks, lunar and martian habitats, and ground vehicles destined for Mars could then be made thinner to reduce weight, saving fuel costs, Odegard says.

Carbon nanotubes (CNTs) could be the answer. Pristine CNTs have fewer defects than carbon fibers, so they are stronger and stiffer. Producing quality nanotubes on a large scale will be key for the composites’ success, Odegard says, but the materials involved are expensive. To reduce the costs of discovery, a NASA-funded consortium of universities and companies, called the Institute for Ultra-Strong Composites by Computational Design (US-COMP), is using simulation and modeling to develop the next generation of ultralight, space-ready composites. Odegard leads the group.

Computational design allows the team to simulate the mechanical properties and interactions of nanotubes, resins, and composites all the way from the quantum level to the macroscale. Then the researchers make and test the most promising candidate materials. As US-COMP searches for the best materials for specific jobs, “relying on computational modeling is less expensive and much faster” than purely experimental methods, Odegard says.

US-COMP’s CNT composites are approaching NASA’s material goals, having reached double the stiffness of state-of-the-art carbon fiber composites used in the aerospace industry today.

Among US-COMP’s ranks is Nanocomp Technologies in Merrimack, New Hampshire, the only company in the U.S. that mass manufactures CNT products. After a series of simulations and tests, the US-COMP team has chosen Nanocomp’s CNT yarns—which are made by bundling hundreds of CNTs into fibers and then twisting or braiding them—as the reinforcement material for the composite. Now the researchers are using simulations to analyze various resins, such as epoxies, cyanide esters, and polybenzoxazines, in search of the right material to complement the nanotube yarns.

“We’re trying to design brand-new materials from scratch,” Odegard says. “The simulations provide good estimations, but ultimately we need laboratory tests for proof of concept.”

At some point, the rubber—or whatever test material—must hit the road. Space is a domain of extremes, and the first trial is right at launch: enormous vibrations and sound waves generated during liftoff can cause significant damage, especially as structural materials get thinner and lighter.

At NASA Glenn, Vivod works on aerogels that can absorb this vibroacoustic energy to keep payloads safe. Aerogels are fine networks built from a solid material such as a metal or a ceramic. Because air makes up most of their volume, they are terrific insulators.

Silica aerogels are already used to insulate batteries and electronics from extreme temperatures on the Mars rovers. Invented in the 1930s, they are made by mixing silica with a solvent to produce a gel and then removing the liquid.

That tricky last step can collapse the fragile gel structure, and researchers have developed new techniques, like supercritical fluid extraction and sublimation, to do it successfully, expanding the realm of substances that chemists can turn into aerogels.

For crewed missions, Vivod is looking at aerogels made of polyimides: polymers with stiff, ring-shaped structures and strong interactions between the nitrogen atoms and carbonyl groups on adjacent polymer chains. These qualities endow polyimides with enough resistance to heat and chemical degradation to replace the films, adhesives, or foams used for aerospace parts.

Aerogels are a new incarnation of polyimides. Polyimide aerogels could find a larger variety of uses than silica aerogels because they are stronger and more flexible, Vivod says, and “you can really tailor the backbone to tweak the chemistry.”

Laboratory tests show that polyimide aerogels are better than the melamine foams that spacecraft currently use to reduce vibrations during launch. “In the launch environment, sound levels are at 160 dB,” Vivod says. “We can bring that down by 50 dB. And then there’s volume savings. A quarter inch [0.6 cm] of polyimide aerogel behaves the same as 4 inches of melamine foam. Plus, you can remove the heavy rubber that’s often used for vibration dampening.”

## VOYAGE

As spacecraft rocket toward the stars, they face a host of other punishing conditions. Flying at blistering speeds creates scorching heat. Outside Earth’s atmosphere, the sun bakes the surface of the spacecraft at temperatures many hundreds of degrees Celsius. In the shadows, temperatures plummet to many hundreds of degrees below zero. Radiation can be a hazard to electronics and astronauts. Then there are debris and micrometeorites to worry about. Micrometeorites, smaller than a grain of sand, travel much faster than the speed of sound and can create minuscule cracks in spacecraft hulls.

Here, too, polyimide aerogels could help, Vivod says. The materials insulate well against extreme heat and cryogenic temperatures. By pinning ultraviolet-absorbing melanin molecules to the polymer backbone and impregnating the material with radiation-scattering nanoparticles, NASA Glenn researchers are making thin, radiation-protecting films for use in martian habitats and space suits.

**Figure:**
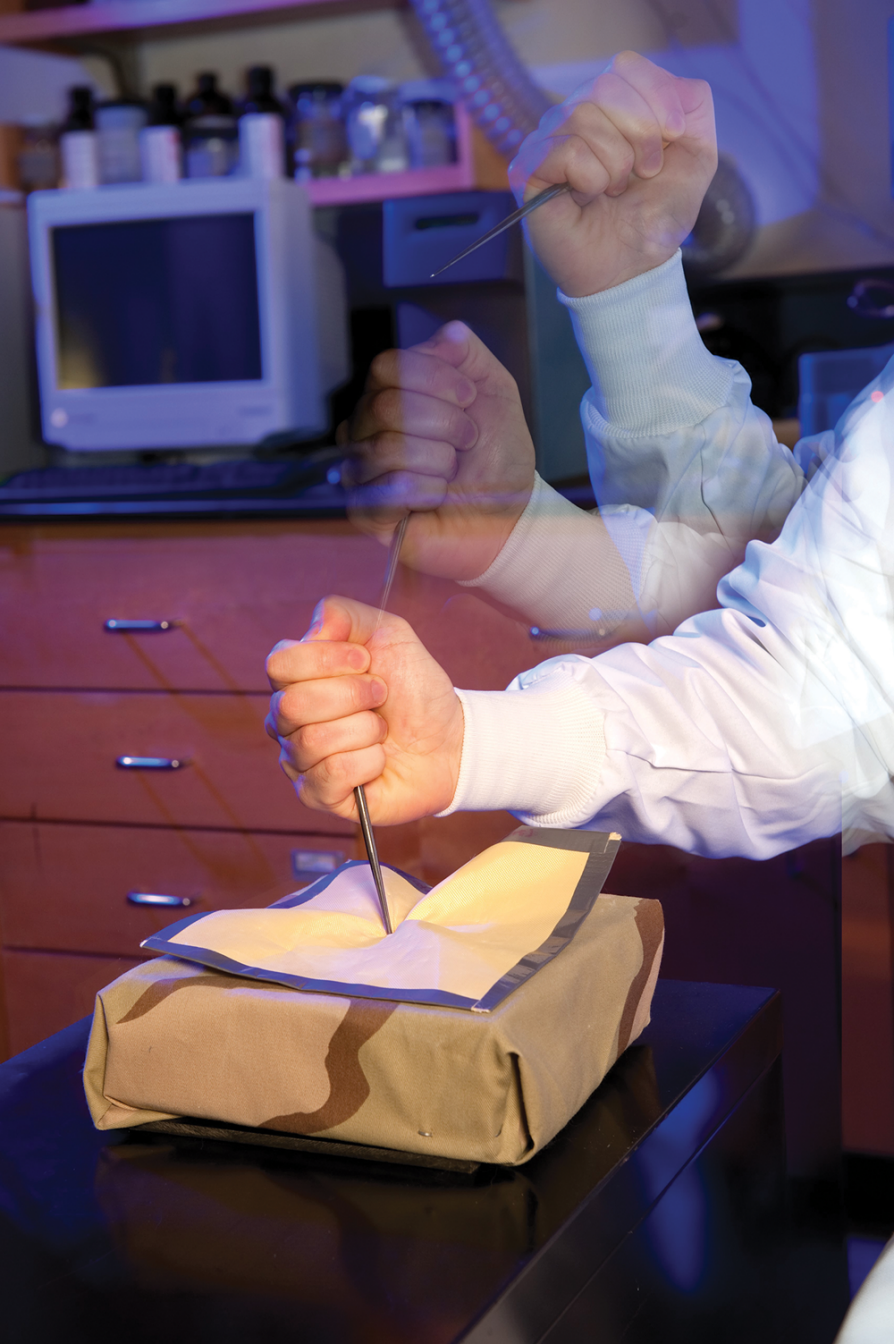
Fabrics soaked in shear-thickening fluids, which solidify under impact, could protect space suits from micrometeorites zooming through space and from sharp martian dust. Credit: Norman J. Wagner.

Researchers at NASA and elsewhere are also taking a fresh look at ceramics for lightweight protection. NASA uses hard, strong ceramics like silicon carbide and alumina to make heat shields, and also combines ceramic fibers with Kevlar, the stuff of bulletproof vests, to make shields that protect against debris and meteorite impact. In November, the agency tested an inflatable heat shield made of silicon carbide fiber cloth: the shield withstood temperatures nearing 1450 °C as it safely returned a payload to Earth.

Cheryl Xu, a mechanical and aerospace engineer at North Carolina State University, is adding nanomaterials to high-temperature ceramics to form multifunctional composites that absorb radiation and are tough and flexible. Infusing silicon carbonitride ceramics with CNTs, for instance, makes the materials able to withstand 1000 °C and flexible enough to bend in half without snapping. And by adding boron nitride nanotubes to ceramics, Xu’s group has made composites that absorb harmful neutron radiation in addition to withstanding searing heat.

Beyond insulating materials that keep warmth in and extreme temperatures out, spacecraft today have complex thermal control systems that pipe excess heat out of the spacecraft into space through radiator panels. That is useful when a crew capsule is in orbit, but during long coasting phases as astronauts barrel toward Mars, systems get shut down, heat generation is minimal, and the environment is frigid.

“What we want is a radiator that in a cold situation curls up and retains warmth and in a hot situation spreads out and cools off,” says Darren Hartl, an aerospace engineer at Texas A&M University. He has turned to shape-memory alloys to make these morphing radiators. The alloys are originally made with a highly stable crystalline structure that they seemingly remember after they’re deformed, he explains. “They will, upon heating, go back to their remembered shape.”

**Figure:**
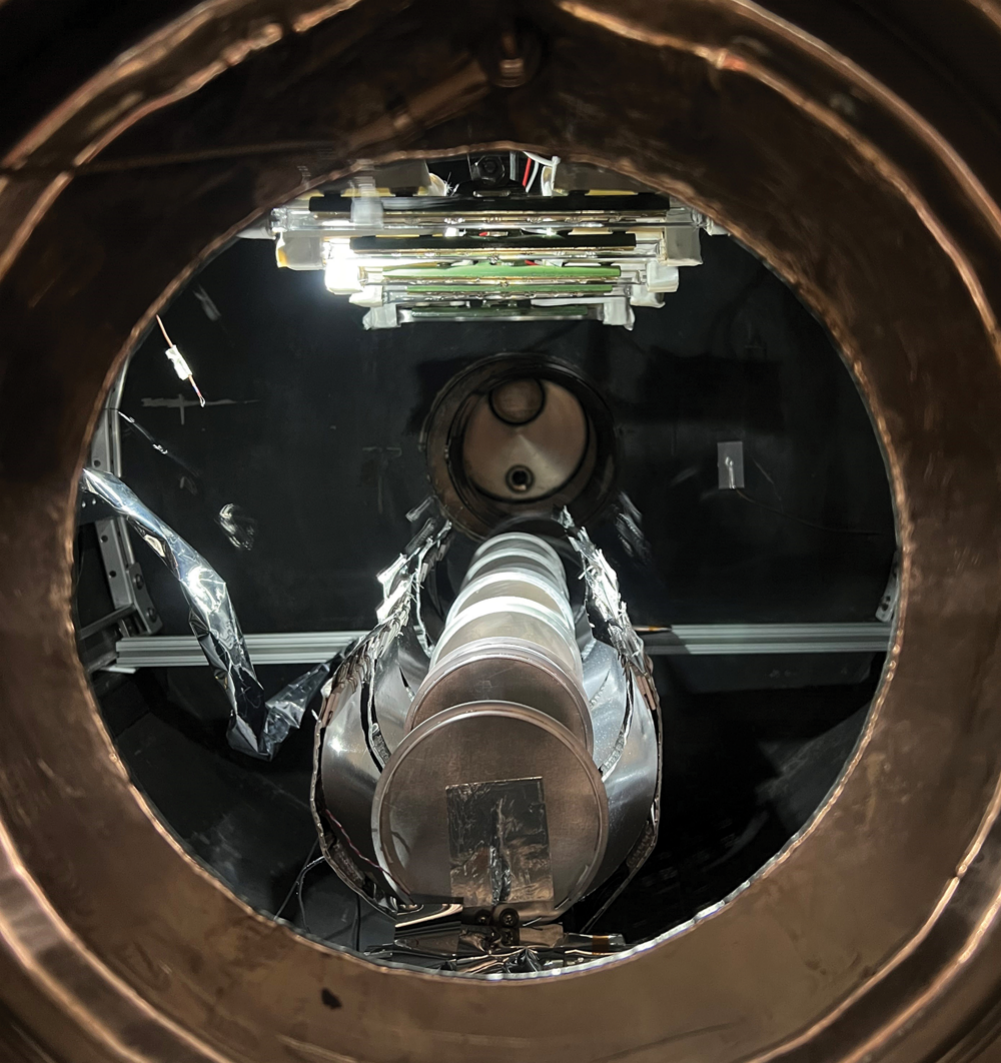
As the temperature of a 3 ft × 3 ft vacuum test chamber changes from −18 to 35 °C, radiator panels made from a shape-memory alloy are triggered to autonomously close and open. Credit: Priscilla Nizio/Texas A&M University.

By adding specialty metals like niobium, zirconium, and palladium to that base recipe, Hartl and colleagues at NASA Glenn have adapted shape-memory alloys to work in space-relevant temperatures. Their curved radiator panels open when the spacecraft needs to let out some heat. A prototype tested in January in a vacuum chamber at NASA’s Lyndon B. Johnson Space Center showed that the radiator panels autonomously closed and opened again as the temperature ranged from −18 to 35 °C.

## EXPLORATION

During space walks, and once on the moon or the Red Planet, space suits will be the only thing between human explorers and their harsh environments. Several advanced materials have gone into NASA’s new Artemis space suits for this reason. The suits’ outermost layer is tough—made of flame-resistant Nomex, waterproof Gore-Tex, and bulletproof Kevlar—“but it’s not particularly good against puncture,” says Norman J. Wagner, a chemical and biomolecular engineer at the University of Delaware.

Tiny, sharp particles in the lunar and martian dust can permeate gaps in the woven fabric. So Wagner is imparting puncture resistance by soaking the fabrics in shear-thickening fluids. These are colloidal suspensions of nanoparticles in carrier fluids that under impact instantly transform from a liquid to a solid-like state. School students learn about the concept by mixing glue and contact solution to make slime, or cornstarch and water to make Oobleck. Wagner says STF Technologies, a start-up he cofounded, has provided materials to Axiom Space, the contractor NASA has chosen to produce Artemis space suits.

With a new NASA grant, his team is now working on a novel material for the outermost layer that could withstand the −230 °C temperatures in the moon’s shadowy craters known to have water ice. The nonwoven material will be made from a polyimide film.

At NASA Glenn, Vivod and her colleagues have also recently discovered that their aerogels can protect against ballistic impact, which could lend itself to use in habitat and suit materials. To test the aerogels, the researchers put blocks of the materials in a vacuum chamber and shot them with 3 mm wide steel pellets, which reached velocities ranging from 200 to 1300 m/s. “Think of it as a bunch of layers of a trapeze net and then launching someone from a cannon at it,” Vivod says. The aerogels were able to absorb at least 20% of impact energy, and even if they’re not yet ready for use in space suits, it’s a start, she says.

For ground vehicles that can traverse rocky, dusty slopes, shape-memory alloys could play an important role in airless tires that can deform drastically but recover their original shape. Materials research engineer Santo Padula at NASA Glenn made the tires by interlocking wires and springs made of shape-memory alloys into a special two-layer pattern.

The tires have undergone rigorous testing for traction on sandy slopes at Glenn’s lunar test facility and at the Jet Propulsion Laboratory’s Mars Yard, which simulates the grueling rocky and sandy terrain of the Red Planet. “The tires deform and envelop obstacles or grip to climb,” Padula says.

**Figure:**
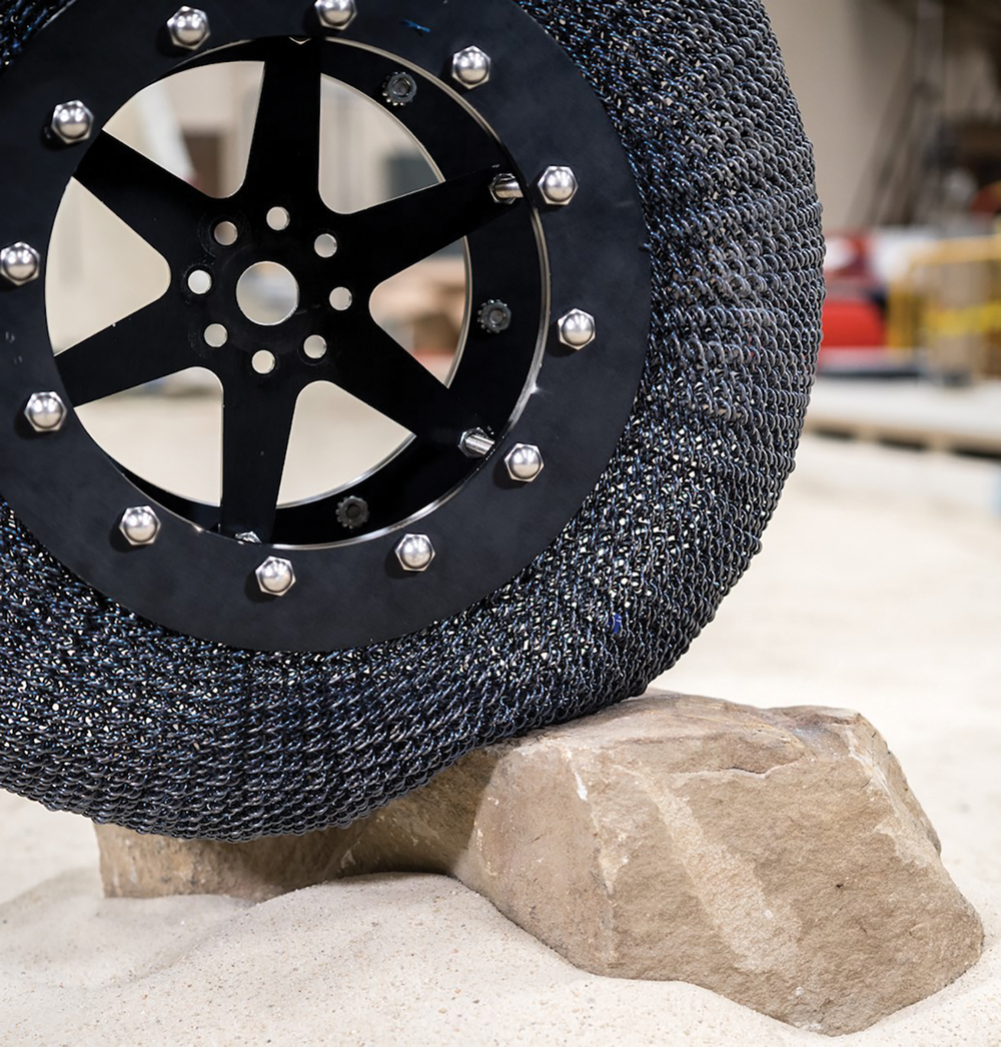
A tire made of interlocked shape-memory alloy wires can grip rocks without getting damaged, making them good for rugged terrain like the surface of Mars. Credit: NASA.

The Glenn team is now focusing on getting these materials from the laboratory to production and use. They are exploring the best metal combinations to reduce cost and assessing durability. Plus, they are developing test standards, a part of new materials technologies that is often overlooked but critical for the technology to get certified for flight.

It’s never too early to think about how scalable and cost-effective an emerging technology is, and exactly where it can be applied, says Emilie Siochi, a materials scientist at NASA’s Langley Research Center. “Decisions on materials are made a long time before launch because of all the testing and certification needed. We need to be able to reduce the time from discovery to use.”

Sometimes, she says, “better” can be the enemy of “good enough.” People designing space mission systems do not care whether a material is “cool,” she says; they care about solving a problem effectively while keeping safety, time, and cost in mind. So while discovery is important, researchers also need to stay grounded with time and budget constraints—and yes, maybe that means picking up a roll of duct tape in a pinch. “Ideas like carbon nanotubes and self-healing materials sound science fictiony. But their performance has to meet a need, and we have to convince somebody there is a business case for it.”

## Prachi Patel is a freelance contributor to

Chemical & Engineering News*, the independent news outlet of the American Chemical Society*.

